# Could the patient have been seen by a nurse; a questionnaire based survey of GP and patient views in Danish general practice

**DOI:** 10.1186/1471-2296-14-171

**Published:** 2013-11-14

**Authors:** Karen Busk Nørøxe, Grete Moth, Helle Terkildsen Maindal, Peter Vedsted

**Affiliations:** 1 Research Unit for General Practice, Department of Public Health, Aarhus University, Denmark, Bartholins Alle 2, Aarhus C 8000, Denmark; 2 Section for Health Promotion and Health Services, Department of Public Health, Aarhus University, Denmark, Bartholins Alle 2, Aarhus C 8000, Denmark

**Keywords:** Denmark, General practice, Doctor-nurse substitution, Patients’ perspective, Chronic conditions, Follow-up consultations, Health service delivery, Patient-doctor-relationship

## Abstract

**Background:**

Nurses in Denmark have been increasingly involved in general practice care, which may have implications for the role of the general practitioner (GP) and patients’ experience of primary care. The aim of this study was to explore possibilities of doctor-nurse substitution seen from GP and patient perspectives and patient preferences in regard to consultations with a personal GP.

**Methods:**

The study was based on data from a Danish survey on disease patterns in general practice (KOS 2008). Background information on patients and GPs was linked with their responses to whether a nurse could have substituted the GP in consultations and patient-assessed importance of seeing a personal GP. Associations were measured with prevalence rate ratio (PR).

**Results:**

Doctor-nurse substitution was a possibility in 14.8% of consultations according to GPs and in 11.7% according to patients. GP and patient agreed on substitution in 3.5% of consultations (Kappa = 0.164). Follow-up consultations were more often feasible for substitution than new episode according to GPs (adj. PR = 2.06 (1.62-2.62)), but not according to patients (adj. PR = 1.02 (0.64-1.33)). Follow-up consultations were related to high importance of seeing the personal GP (adj. PR = 1.18 (1.05-1.33). For both patients and GPs, consultations with patients with chronic conditions were not significantly associated with nurse substitution. Male and younger patients did more often suggest substitution than women and older patients. For GPs, increasing patient age was associated with relevance of substitution. Patients who found it 'very important’ to see their personal GP were less likely to consider nurse substitution a possibility (adj. PR = 0.57 (0.45-0.71).

**Conclusions:**

GPs and patients found nurse substitution relevant in more than one in ten consultations, although they rarely agreed on which consultations. Follow-up consultations and consultations with older patients were associated with GPs considering nurse substitution appropriate more often. For patients, male and younger patients most often found substitution relevant. High importance of seeing the personal GP may contribute to patient reluctance to nurse substitution, especially for follow-up consultations. The results indicate a need for involving patients’ perspective when altering the future roles of primary health care professionals.

## Background

The demands for primary care services are currently increasing in many countries due to demographic changes with population ageing, increasing prevalence of chronic conditions, and continuous expansion of medical knowledge accompanied by new options for diagnosing, prevention and treatment of medical conditions. In a primary-based health care system as the Danish, where the majority of all health-related contacts are handled in primary care, the increasing demands have led to increased workload for general practitioners (GPs) [1]. This has prompted a rethinking of the roles of health care professionals and the organisation of care delivery in order to achieve efficient, cost-effective and high-quality primary care services. A much debated issue is the substitution of the GP by a nurse. However, knowledge is lacking on the implications in a Danish healthcare context.

Internationally, the role of nurses has developed in different directions. In many countries, primary care nurses are no longer simply regarded as assistants for GPs, but are seen as independent healthcare providers licensed as *Nurse Practitioners*[2,3]. In other countries, including Denmark, the role of the primary care nurse has expanded to include practice management under the direction of a physician. Danish primary care has been characterized by an increased use of nurses during the previous decades [4,5]. Since 2003, Danish GPs have been remunerated for consultations managed by clinical staff without involvement of the GP, and increased use of practice personnel, including licensed nurse practitioners, is recommended by the Danish Ministry of Health [6].

A recent Danish study indicated that nurses tend to undertake other tasks than GPs; hence the nature of nurse-led care may be seen as complementary rather than substitutive to the GP [7]. If the role of the nurse is to be further expanded, the possible impact on the doctor-patient relationship should be addressed and considered when determining future tasks and responsibilities, in particular since the doctor-patient relationship is considered important by patients.

The aim of this study was (i) to explore the possibilities of doctor-nurse substitution from the GPs’ and the patients’ point of views and (ii) to describe patient preference in regard to consultations with a personal GP.

## Methods

### Design, setting and study population

The study is based on data from ’Survey of Reasons for Encounter and Disease Patterns in General Practice’ (KOS 2008), which is a large survey regarding patient contacts to general practice in the Central Denmark Region. The region has 1.2 million inhabitants, approximately equivalent to 20% of the Danish population, and covers 13% of the GPs [1,8]. All 871 GPs in the region were invited to participate. Registered information on the GPs and their clinics included gender, number of years in general practice (seniority), type of practice (single-handed or group practice) and whether one or more nurses were employed. The study methods have previously been described elsewhere [1].

During a 12-month period (from December 2008 to December 2009), the participating GPs registered all their patient contacts on a randomly assigned date. For each patient contact, the GP was asked to fill in a one-page registration form covering a range of questions, e.g. on chronic disease, reason for encounter, new episode or follow-up, diagnosis, perceived burden of the consultation, referral to specialist care, suspicion of serious disease and whether a nurse could have substituted the GP. Diagnostic information was classified according to the International Classification of Primary Care (ICPC) [9]. Most registrations were made immediately after the contact and all within the day of the contact.

Questionnaires were subsequently sent to patients for each registered contact in general practice covering issues of their experience of the contact and of their health, including the question 'Could the contact have been with a nurse instead?’ with the response categories 'Yes’, 'No’ and 'Don’t know’. Patients who consulted their personal GP were also asked how important they rated the contact with their personal GP (in contrast to a random GP) with the following response categories: 'Very important’, 'Somewhat important’, 'Not very important’, 'Not important’, 'Don’t know’. In the analyses, these were dichotomised into 'Very important’ and 'Not very important’ with the latter including all other response categories than 'Very important’. The majority of patients received the questionnaire one week after the contact. However, some patients received it later due to delay in return of GP-registrations. Patients who had not returned the questionnaire within two weeks received a reminder with a new questionnaire.

### Data

Data included all GP-patient contacts restricted to adults (aged ≥18 years). Patients in contact with the clinical staff only were not included. Telephone contacts, email contacts, home visits and consultations due to prophylactic issues or issuance of certificates were excluded.

**Figure 1:**
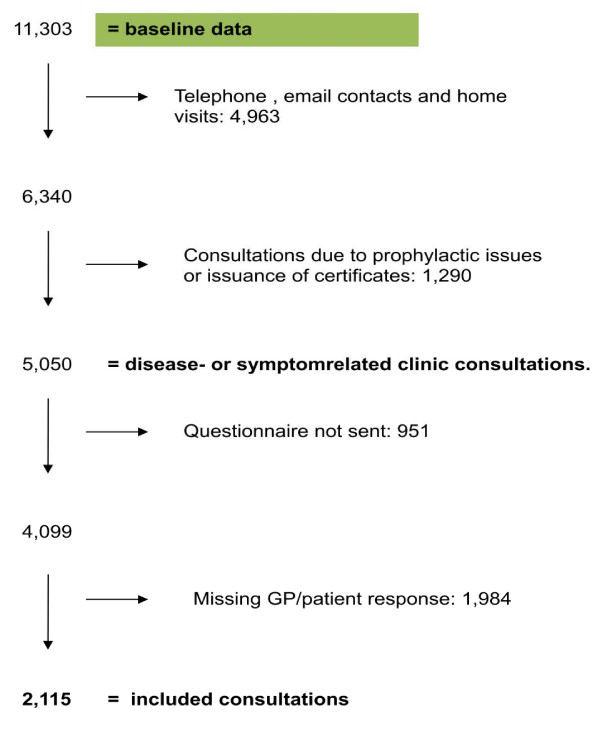
Flow chart depicting process for inclusion of consultations in the study.

Patients did not receive a questionnaire if the GP had not stated the patient’s civil registration number or in case of publicly recorded protection against participation in research, earlier participation in the study or death. All contacts with missing GP or patient response were excluded (Figure 1).

Patient age was categorised into five groups (18–40, 41–50, 51–60, 61–70 and >70 years), and a variable of multi-morbidity was generated based on GP statements of chronic conditions (0, 1, ≥2 chronic conditions). GP seniority was categorized into four groups (<6, 6–10, 11–20, >20 years as a GP).

### Analysis

For all consultations and for subgroups, we calculated the share of GPs and the share of patients who found that a nurse could have substituted the GP. ‒square tests were used to examine the differences between groups. Possibility of nurse substitution according to GPs and patients and the association with characteristics of the consultations were analysed with a generalized linear model (GLM) and adjusted for patient-related factors (gender, age, number of chronic conditions registered by the GP and type of contact (new episode or follow-up)) and GP -related factors (gender, seniority and type of practice). For patients who consulted their personal GP (in contrast to a random GP), the association between 'high importance’ of seeing the personal GP and patient-related factors was also analyzed with a generalized linear model (GLM) and adjusted for the above-mentioned patient-related factors as well as patient attitude towards nurse substitution. Associations were calculated as the prevalence difference (PD) and the prevalence ratio (PR) with 95% confidence intervals (95% CI). For all consultations and subgroups, the agreement of nurse substitution between GPs and patients was examined by Kappa statistics. Analyses were performed in STATA 11.

### Ethics

The project was approved by the Danish Data Protection Agency (J.no. 2008-41-2195) and by the Danish Health and Medicines Authority (J.no. 7-604-04-2/49/EHE). According to Danish law, approval by the National Committee on Health Research Ethics was not required as no biomedical intervention was performed. The participating GPs received remuneration that was partly dependent on the number of registered contacts.

## Results

A total of 404 (46.6%) of the invited GPs participated in this study. A comparison between participants and non-participants on type of clinic and the distribution of listed patients showed no statistically significant differences. Male GPs more often participated compared to female GPs (55.4% vs. 44.6%), and the share of GPs with more than 20 years of professional seniority was lower among participants than among the background group of GPs (20.4% vs. 25.5%). Information on whether one or more nurses were employed in the practice was obtained for 301 of the participating GPs. Of these, 90.0% had at least one nurse employed.

A total of 2,115 GP consultations were included in the analyses (Figure 1). Analysis of non-response among patients showed no gender differences (p = 0.133). Fewer younger patients (aged 18–40 years) responded (23.1% vs. 39.0%), whereas patients aged 61–70 responded more often (21.9% vs. 12.3%) (p < 0.001).

Table 1 shows the number and proportion of consultations suitable for nurse substitution according to GPs and patients. Overall, GPs found that 14.8% and patients that 11.7% of all consultations could have been completed by a nurse instead of the GP (p = 0.013).

**Table 1:** Consultation type and proportion of patients and GPs in favour of nurse substitution

		**Consultations**	**GP states “YES”**	**Patient states “YES”**	**P-value**
		**N (%)**	**N (%)**	**N (%)**	
**Patient characteristics**					
*Gender*	**Female**	1,319 (62.4)	179 (13.6)	137 (10.4)	0.010
	**Male**	795 (37.6)	121 (15.2)	109 (13.7)	0.392
	**No inf.**	1 (0.05)			
*Age (years)*	**18-40**	482 (22.8)	48 (10.0)	96 (19.9)	<0.001
	**41-50**	370 (17.5)	33 (8.9)	36 (9.7)	0.704
	**51-60**	389 (18.4)	45 (11.6)	30 (7.7)	0.068
	**61-70**	467 (22.1)	95 (20.3)	51 (10.9)	<0.001
	**>70**	406 (19.2)	78 (19.2)	32 (7.9)	<0.001
	**No inf.**	1 (0.1)			
*Number of chronic conditions*	**0**	860 (40.7)	95 (11.1)	115 (13.3)	0.141
	**1**	692 (32.7)	118 (17.1)	77 (11.1)	0.002
	**>** **2**	563 (26.6)	87 (15.5)	54 (9.6)	0.003
*Type of contact*	**New episode**	1,031 (48.7)	94 (9.1)	125 (12.1)	0.027
	**Follow-up**	967 (45.8)	189 (19.5)	107 (11.1)	<0.001
	**No inf.**	117 (5.5)			
Patient-assessed importance of seeing the personal GP*	**Very important**	1,230 (60.0)	159 (12.9)	77 (6.2)	<0.001
	**Not very important**	642 (35.0)	111 (16.6)	131 (20.5)	0.154
	**No inf.**	107 (5.0)			
**GP characteristics**					
*Gender*	**Female**	1,207 (57.1)	104 (11.5)	105 (11.6)	0.941
	**Male**	906 (42.8)	195 (16.2)	140 (11.6)	0.001
	**No inf.**	2 (0.1)			
*Seniority (years in practice)*	**<6**	440 (20.8)	69 (15.6)	68 (15.5)	0.926
	**6-10**	281 (13.3)	34 (12.1)	23 (8.2)	0.124
	**11-20**	701 (33.1)	99 (14.1)	86 (12.3)	0.305
	**>20**	693 (32.8)	98 (14.1)	69 (10.0)	0.170
*Type of practice*	**Single-handed**	555 (26,2)	86 (15.5)	75 (13.5)	0.348
	**Group**	1,476 (69.8)	200 (13.5)	164 (11.1)	0.044
	**No inf.**	84 (4.0)			
**All consultations**		2,115 (100)	300 (14.8)	246 (11.7)	0.013

*1872 patients who consulted their personal GP.

GPs were statistically significantly more likely than patients to suggest nurse substitution when the patients were female, aged 61 years or more, had chronic conditions, assessed seeing personal GP 'very important’ , the consultation was a follow-up encounter and when the GP was a male. GPs more often found consultations with patients with chronic conditions feasible for nurse substitution, whereas the patients expressed the opposite. In general, patients were less likely than the GPs to suggest nurse substitution with the exception of patients below 40 years of age (19.9% vs. 10.0%, p < 0.001) or when consulting for a new episode (12.1% vs. 9.1%, p = 0.027). Significant differences between GPs and patients were found in the assessment of nurse substitution for different patient age groups and in opposite directions for GPs and patients.

In 3.5% of all consultations, GPs and patients agreed upon nurse substitution, whereas in 77.7% of the consultations they agreed that substitution was not a possibility (Kappa = 0.164). GPs and patients more often agreed on substitution as a possibility when the patient was male (Kappa = 0.207), the consultation was a follow-up encounter (Kappa = 0.190) and the GP was working in single-handed practices (Kappa = 0.238) (data not shown).

A positive patient attitude towards nurse substitution (Table 2) was statistically significantly associated with being a male patient (adj. PR = 1.42 (1.10-1.82)) and with age groups under 40 years of age (p ≤ 0.001).

**Table 2:** Association between contacts involving a positive patient attitude towards nurse substitution and characteristics related to patients and GPs

		**Patients assessing nurse substitution feasible**				
		**Prevalence (N)**	**Prevalence difference (95% CI)**	**Prevalence ratio (95 CI)**	Adj. prevalence ratio* (95 CI)	**p-value**
**Patient characteristics**						
*Gender*	**Female**	0.10 (137)	ref	1	1	
	**Male**	0.14 (109)	0.03 (0.004-0.06)	1.32 (1.04-1.67)	1.42 (1.10-1.82)	0.007
*Age (years)*	**18-40**	0.20 (96)	ref	1	1	
	**41-50**	0.10 (36)	-0.10 (-0.15- -0.06)	0.49 (0.34-0.70)	0.46 (0.31-0.67)	<0.001
	**51-60**	0.08 (30)	-0.12 (-0.17- -0.08)	0.39 (0.26-0.57)	0.34 (0.22-0.53)	<0.001
	**61-70**	0.11 (51)	-0.09 (-0.14- -0.04)	0.54 (0.40-0.75)	0.55 (0.39-0.78)	0.001
	**>70**	0.08 (32)	-0.12 (-0.16- -0.08)	0.39 (0.27-0.58)	0.46 (0.30-0.70)	<0.001
*Number of chronic conditions*	**0**	0.13 (115)	ref	1	1	
	**1**	0.11 (77)	-0.02 (-0.06- 0.01)	0.83 (0.63-1.09)	0.96 (0.72-1.30)	0.799
	**≥2**	0.10 (54)	-0.04 (-0.07- -0.005)	0.71 (0.52-0.97)	0.92 (0.64-1.33)	0.655
*Type of contact*	**New episode**	0.12 (125)	ref	1	1	
	**Follow-up**	0.11 (107)	-0.01 (-0.04-0.02)	0.91 (0.72-1.16)	1.02 (0.64-1.33)	0.896
**GP characteristics**						
*Gender*	**Female**	0.12 (105)	ref	1	1	
	**Male**	0.12 (140)	0.0001 (0.027-0.03)	1.00 (0,79-1.27)	0.98 (0.75-1.28)	0.863
*Seniority (years in practice)*	**<6**	0.15 (68)	ref	1	1	
	**6-10**	0.08 (23)	-0.07 (-0.12- -0.03)	0.53 (0.34-0.83)	0.62 (0.39-0.97)	0.038
	**11-20**	0.12 (86)	-0.03 (-0.07- -0.01)	0.79 (0.59-1.07)	0.87 (0.64-1.19)	0.380
	**>20**	0.10 (69)	-0.05 (-0.10- -0.01)	0.64 (0.47-0.88)	0.73 (0.53-1.04)	0.082
*Type of practice*	**Group**	0.11 (164)	ref	1	1	
	**Single-handed**	0.14 (75)	0.02 (-0.01-0.06)	1.22 (0.94-1.57)	1.25 (0.94-1.57)	0.090

*Adjusted for patient related factors (gender, age, number of chronic conditions, type of contact (new episode or follow up)), and GP related factors (gender, number of years as GP, type of practice).

GPs’ positive attitude towards nurse substitution (Table 3) was statistically significantly associated with older patient age (aged 61–70 years and >70 years) (adj. PR = 1.71 (1.20-2.44) and adj. PR = 1.67 (1.14-2.44)) and follow-up encounters (adj. PR = 2.06 (1.62-2.62)). Male GPs had a statistically higher likelihood of suggesting nurse substitution(adj. PR = 1.40 (1.09-1.80)). Chronic conditions were not statistically significantly associated with substitutability for neither GPs nor patients in the adjusted analyses.

**Table 3:** Association between contacts involving a positive GP attitude towards nurse substitution and characteristics related to patients and GPs

		**GPs assessing nurse substitution feasible**				
		**Prevalence (N)**	**Prevalence difference (95% CI)**	**Prevalence ratio (95 CI)**	Adj. prevalence ratio* (95 CI)	**P-value**
**Patient characteristics**						
*Gender*	**Female**	0.14 (179)	ref	1	1	
	**Male**	0.15 (121)	0.02 (-0.02-0.05)	1.12 (0.91-1.39)	0.89 (0.71-1.12)	0.310
*Age (years)*	**18-40**	0.10 (48)	ref	1	1	
	**41-50**	0.10 (33)	-0.01 (-0.05- 0.03)	0.90 (0.59-1.37)	0.80 (0.51-1.26)	0.336
	**51-60**	0.13 (45)	0.02 (-0.03- 0.06)	1.16 (0.79-1.71)	0.99 (0.66-1.48)	0.941
	**61-70**	0.21 (95)	0.10 (0.09-0.15)	2.04 (1.48-2.82)	1.71 (1.20-2.44)	0.003
	**>70**	0.20 (78)	0.09 (0.05-0.14)	1.93 (1.38-2.70)	1.67 (1.14-2.45)	0.009
*Number of chronic conditions*	**0**	0.11 (95)	ref	1	1	
	**1**	0.17 (118)	0.06 (0.03- 0.10)	1.54 (1.20-1.99)	1.21 (0.92-1.60)	0.179
	**≥2**	0.15 (87)	0.04 (0.01- 0.08)	1.40 (1.07-1.83)	0.91 (0.66-1.25)	0.569
*Type of contact*	**New episode**	0.09 (94)	ref	1	1	
	**Follow-up**	0.20 (189)	0.10 (0.07-0.14)	2.14 (1.70-2.70)	2.06 (1.62-2.62)	<0.001
**GP characteristics**						
*Gender*	**Female**	0.12 (104)	ref	1	1	
	**Male**	0.16 (195)	0.05 (0.02-0.08)	1.41 (1.13-1.76)	1.40 (1.09-1.80)	0.009
*Seniority (years in practice)*	**<6**	0.16 (69)	ref	1	1	
	**6-10**	0.12 (34)	-0.04 (-0.09-0.02)	0.77 (0.53-1.13)	0.82 (0.56-1.20)	0.307
	**11-20**	0.14 (99)	-0.02 (-0.06-0.03)	0.90 (0.68-1.20)	0.81 (0.60-1.08)	0.152
	**>20**	0.14 (98)	-0.02 (-0.06-0.03)	0.90 (0.68-1.20)	0.76 (0.56-1.04)	0.090
*Type of practice*	**Group**	0.16 (86)	Ref	1	1	
	**Single-handed**	0.14 (200)	0.02 (-0.02-0.05)	1.14 (0.91-1.44)	1.07 (0.85-1.37)	0.560

*Adjusted for patient-related factors (gender, age, number of chronic conditions, type of contact (new episode or follow-up)), and GP-related factors (gender, GP seniority, type of practice).

High importance of seeing the personal GP (Table 4) was significantly associated with follow-up consultations (adj. PR = 1.18 (1.05-1.33)) and patients not considering substitution a possibility (adj. PR = 0.57 (0.45-1.33). High importance of seeing the personal GP was also found to be associated with female sex, patients aged over 40 years and chronic conditions, but in the unadjusted analyses only.

**Table 4:** Prevalence ratios of the patient-assessed importance of seeing the personal GP (very important as the response variable) in consultations with the personal GP, adjusted for the presented characteristics

		**Patients assessing the encounter with the personal GP 'very important’**				
		**Prevalence (N)**	**Prevalence difference (95% CI)**	**Prevalence ratio (95 CI)**	**Adj. prevalence ratio (95 CI)**	**P-value**
**Patient characteristics**						
Substitution considered a possibility	Yes	0.37 (82)	ref	1	1	
	No	0.70 (1,246)	-0.32 (-0.39- -0.26)	0.54 (0.45-0.64)	0.57 (0.45-0.71)	<0.001
*Gender*	Female	0.70 (867)	ref	1	1	
	Male	0.61 460)	-0.09 (-0.13- -0.05)	0.87 (0.81-0.93)	0.87 (0.77-1.03)	0.017
*Age (years)*	18-40	0.56 (234)	ref	1	1	
	41-50	0.69 (233)	0.13 (0.06-0.20)	1.24 (1.11-1.38)	1.17 (0.97-1.42)	0.099
	51-60	0.68 (248)	0.12 (0.05- 0.19)	1.21 (1.08-1.35)	1.11 (0.92-1.35)	0.276
	61-70	0.73 (335)	0.16 (0.10-0.23)	1.29 (1.17-1.43)	1.20 (1.00-1.44)	0.053
	>70	0.66 (278)	0.10 (0.04-0.17)	1.18 (1.06-1.32)	1.04 (0.82-1.26)	0.711
*Number of chronic conditions*	0	0.59 (449)	ref	1	1	
	1	0.69 (468)	0.10 (0.05- 0.15)	1.16 (1.08-1.26)	1.10 (0.96-1.27)	0.174
	≥2	0.72 (411)	0.13 (0.08- 0.18)	1.22 (1.13-1.32)	1.14 (0.97-1.33)	0.108
*Type of contact*	New episode	0.73 (705)	ref	1	1	
	Follow-up	0.60 (548)	0.13 (0.09-0.17)	1.22 (1.14-1.30)	1.18 (1.05-1.33)	0.004

## Discussion

### Main findings

In general, GPs more often suggested nurse substitution than patients (15% vs. 12%). GPs and patients agreed on substitution as a possibility in only 3.5% of all consultations, implying that the agreement was low on nurse substitution of the GP. Male and younger patients had a higher likelihood of suggesting nurse -substitution. Among GPs, patient age over 60 years, follow-up consultations and male sex of the GP increased the likelihood to suggest nurse substitution. Interestingly, nurse substitution was not associated with having a chronic disease. Patients who did not consider nurse substitution a possibility were significantly associated with high importance of seeing the personal GP compared to seeing a random GP. Seeing the personal GP was more often of high importance for follow-up consultations. A trend towards high importance was also found for female patients, older patients and patients with chronic conditions.

### Strengths and weaknesses

This study provides high statistical precision as half of the GPs in the region (13% of all GPs in Denmark) participated in the study, thus registering more than 10,000 contacts. The GP and patient responses to the possibility of nurse substitution provided paired information on specific consultations in Danish general practice. Therefore, the data represents GP and patient perspectives on the possibility of doctor-nurse substitution for real-life GP consultations and not simply hypothetical assessments of nurse substitution.

The patients’ assessment of nurse substitution may have been affected by the fact that they were actually seen by a GP and hence may have considered this approach proper. However, as this was the case for all patients, this explanation does not account for the variations within subgroups. Further, this implies that our results may underestimate how often a nurse may substitute the GP. Prior experience with consulting a nurse may have an impact on the attitude towards nurse led care. However, we had no data to elucidate this hypothesis.

The GP participation was high. Thus, selection bias can be regarded as low. Still, the non-participating GPs may have represented a group of GPs with a different contact pattern. However, we have no data to support this or the direction of a possible selection bias. Missed registration of chronic conditions by the GPs may have led to dilution of differences between patients with and without chronic conditions.

In general, generalisation to other countries should be made with caution because of international differences in nurse qualifications as well as in the structure of primary care. When interpreting the results of this study it should be held in mind that “GP-nurse-substitution” refers to a specific consultation. Patients and GPs were asked if a nurse could have taken over this particular consultation and not whether the nurse could overtake future aspects of care. In that context, “substitution” should not be seen in contrast to collaborative care which is different from substitution.

### Discussion of results

The more positive attitude towards nurse substitution among male patients may be related to the general finding that women tend to consider the relationship with their GP of higher importance. The relationship with the GP may also be more well-established among female patients as women see the GP more often than men throughout life [1,10]. In contrast to our findings, a Scottish study found female patients to have the most positive attitude towards nurse-led hypothetical encounters [11]. This difference may be related to the fact that Scottish patients are more familiar and therefore more confident with nurse-led care or may be explained by the hypothetical nature of the Scottish study in contrast to the real-life encounters of our study.

GP and patient assessment of substitutability in relation to age is noteworthy. The positive approach to substitution among younger patients may be related to better health, short or no medical history and therefore low need for continuity and interpersonal relations with their GP. A Norwegian study found a similar low need for GP-patient continuity among younger patients [12]. In contrast, the increasing significance of GP-assessed nurse substitutability with increasing patient age may be partly explained by age-related differences in reasons for encounter, e.g. more encounters concerning follow-up of long-term conditions with increasing age. Also, better knowledge of older patients and their medical history may lead to easier medical decisions and facilitate delegation of tasks to nurses. The GPs’ positive approach to nurse led management of follow-up consultations was expected as delegation of the follow-up task is preceded by the GP’s opportunity of medical evaluation, diagnosis and treatment planning.

Care management of patients with chronic conditions is an area characterized by the expanding role of nurses during recent years. Disease management programmes with fixed schedules for frequency and contents of consultations have been developed, and consultations with patients with chronic conditions are considered obvious domains for involvement of practice nurses [13]. However, we found a remarkable disagreement on this between GPs and patients. Adjustment for confounding in our study revealed that GPs would more often recommend the nurse to conduct the follow-up consultations, which was definitely not the case for patients. This latter finding may be related to the importance of continuity of care, which has been shown to be particularly valued by people with long-standing health problems [2].

The finding that patients opposing nurse substitution more often considered seeing their personal GP as 'very important’ compared to patients supporting substitution was expected, but still noteworthy. High importance of seeing the personal GP may be a contributory cause of patients’ reluctance towards substitution and hence may partially explain the divergent opinions among GPs and patients on substitution regarding e.g. follow-up consultations. Our findings indicate that nurse-GP substitution is not only about choosing between persons with different professions, but also concerns the patient’s relationship with the GP and/or a wish for continuity of care. This is in line with a British study showing that the interpersonal continuity of care was important to patients and that replacement of the usual GP with a nurse was acceptable, but only as an exceptional case [14]. Another study showed that some patients feared a worsening of the patient-doctor relationship as a consequence of the increased involvement of nurses [13].

Continuity of care and a close patient-doctor relationship are related and considered important [15,16]. The inter-personal doctor-patient continuity of care is favoured by the Danish primary care system and is highly valued by patients [17]. Even though it could be argued that the relational continuity might as well be between patient and nurse in some cases, the potential impact of substitution on continuity and the doctor-patient relationship should be borne in mind.

When evaluating and developing the roles of primary health care professionals the patient perspective must also be included. Studies have shown high levels of patient satisfaction with nurse-led consultations [2]. However, patient perceived satisfaction with a nurse-led consultation cannot equate with patients’ attitude to the increasing role of nurse led patient care in primary care. Therefore, we need more knowledge about the reasons behind patients’ attitude towards consulting a nurse, including patients’ knowledge of the qualifications of nurses, and experience with consulting a nurse. Further, there seems to be a need for knowledge about increased nurse involvement in primary care and appreciation of the doctor-patient relationship, and also to investigate whether increased nurse involvement have an actual impact on the doctor-patient relationship.

## Conclusion

GPs and patients rarely agreed on consultations where the GP could have been substituted by a nurse. A considerable association was found between nurse substitution and patient age, but in opposite directions for GPs and patients; patients under 40 years of age were significantly more inclined to see a nurse compared to older patients, whereas GPs more often found consultations with patients over 60 years of age suitable for substitution. Moreover, male patients and male GPs were more positive towards nurse substitution compared to women and female GPs. The GPs considered follow-up consultations appropriate for nurse substitution, which was not the case for the patients. For follow-up consultations, patients more often found it 'very important’ to see their personal GP. Thus, the patients’ perspective should be taken into consideration in the delegation of clinical tasks.

## Competing interests

The authors declare that they have no competing interests.

## Authors’ contributions

All authors fulfil the criteria of authorship according to the Vancouver rules for authorship. KBN and GM drafted this paper in close co-operation with HTM and PV. All authors were involved in interpretation of the results and editing of the manuscript. The study was designed by GM and PV. All authors read and approved the final manuscript.

## Pre-publication history

The pre-publication history for this paper can be accessed here:

http://www.biomedcentral.com/1471-2296/14/171/prepub

## Declaration

Funding: This project has been supported by the Central Denmark Region (formerly the County of Aarhus) and the Danish National Research Foundation for Primary Care.
